# Advanced Synthesis and Characterization of CdO/CdS/ZnO Heterostructures for Solar Energy Applications

**DOI:** 10.3390/ma17071566

**Published:** 2024-03-29

**Authors:** Yana Suchikova, Sergii Kovachov, Ihor Bohdanov, Zhakyp T. Karipbayev, Yaroslav Zhydachevskyy, Anastasiia Lysak, Vladimir Pankratov, Anatoli I. Popov

**Affiliations:** 1 The Department of Physics and Methods of Teaching Physics, Berdyansk State Pedagogical University, 71100 Berdyansk, Ukraine; yanasuchikova@gmail.com (Y.S.); essfero@gmail.com (S.K.); naukabdpu@gmail.com (I.B.); zhydach@ifpan.edu.pl (Y.Z.); alysak@ifpan.edu.pl (A.L.); 2 Faculty of Physics and Technical Sciences, L.N. Gumilyov Eurasian National University, Astana 010008, Kazakhstan; karipbayev_zht_1@enu.kz; 3 Institute of Solid State Physics, University of Latvia, 8 Kengaraga, 1063 Riga, Latvia; 4 Institute of Physics, Polish Academy of Sciences, al. Lotnikow 32/46, 02-668 Warsaw, Poland

**Keywords:** CdO/CdS/ZnO heterostructure, electrochemical deposition, oxygen annealing, SEM, EDX, Raman, XRD, photoluminescence

## Abstract

This study introduces an innovative method for synthesizing Cadmium Oxide /Cadmium Sulfide/Zinc Oxide heterostructures (CdO/CdS/ZnO), emphasizing their potential application in solar energy. Utilizing a combination of electrochemical deposition and oxygen annealing, the research provides a thorough analysis of the heterostructures through scanning electron microscopy (SEM), energy-dispersive X-ray (EDX) spectroscopy, X-ray diffraction (XRD), Raman spectroscopy, and photoluminescence (PL) spectroscopy. The findings reveal a complex surface morphology and a composite structure with significant contributions from hexagonal CdS and cubic CdO phases. The study highlights the uniformity in the distribution of luminescent centers and the crystalline quality of the heterostructures, which is evident from the PL analysis. The redshift observed in the emission peak and the additional peaks in the excitation spectrum indicate intricate optical properties influenced by various factors, including quantum confinement and lattice strain. The research demonstrates these heterostructures’ potential in enhancing solar cells’ efficiency and applicability in optoelectronic devices. This comprehensive characterization and analysis pave the way for future optimization and application in efficient and sustainable solar energy solutions.

## 1. Introduction

Over the last few decades, the global energy landscape has undergone transformative changes with a concerted push towards decarbonization and sustainable energy solutions [1,2,3]. Solar energy is at the forefront of this transition [4,5,6]. However, harnessing solar energy with higher efficiency and at lower costs remains a challenge that necessitates continual improvements in materials and technologies [7,8,9,10].

Significant progress in solar cell technology has recently been made as researchers have explored various materials to enhance their efficiency and durability [11,12,13]. Intensive investigations have been conducted into mono-, bi-, and tri-component materials [14,15,16], and methods for creating heterostructures for solar cells are being actively developed [17,18,19]. Among these materials, CdO and CdS have emerged as promising candidates, particularly in dye-sensitized solar cells (DSSC) [20,21]. For instance, CdS has been recognized for its utility in DSSC, and its synthesis primarily revolves around methods such as chemical bath deposition, vacuum evaporation, or sputtering [22,23,24]. Thanks to its unique properties, this material is a bridge in CdTe-based photoelectric cells. It is an intermediate layer between the transparent conductive oxide (TCO) and CdTe layers [25,26].

On the other hand, CdO has been highlighted for its potential application as a transparent conductive oxide (TCO) layer in solar cells [27,28]. An intriguing aspect of CdO is its ability to be deposited as thin films during air annealing, leading to a thin CdO layer [29,30]. Furthermore, in solar cells, the CdO layer in p-Si/CdO, doped with elements like Sn, Sb, or Se, has shown promising results [31].

Today, numerous methods exist for creating heterostructures comprising layers of different semiconductors. Specifically, the sided synthesis technique has been employed to create multi-component nanostructures. Using this method, researchers have demonstrated the formation of CdS and CdSe structures with distinct rod and tetrapod morphologies. The complex designs resulting from this method underscore its potential in creating intricate nanostructures [32]. Hydrothermal techniques have synthesized pine-needle-like CdS/CdO nanocomposites [33].

Alternatively, combining thermal treatment with doping concentration offers a more rational approach. This method has synthesized Cu-doped Cd(OH)_2_-CdO nanostructures, demonstrating how a balance between thermal processes and material doping can yield desirable outcomes [34].

The successive ionic layer adsorption and reaction (SILAR) method utilizes sequential ionic layer adsorption and reaction to achieve its results. A notable discovery using this technique has been the synthesis of the Cd_x_Te_y_O_z_/CdS/ZnO heterostructure, which highlights the importance of the CdS film as a buffer layer [35]. These methods have repeatedly demonstrated their effectiveness in creating heterostructures; however, for scalability and synthesis at industrial levels, it is crucial to develop cost-effective methods that are quick to implement and do not require a vacuum or high-tech equipment. In this aspect, electrochemical methods have shown promise, allowing for surface nanostructuring [36,37,38] or the deposition of films and nanostructures with various morphological configurations [39,40,41]. By adjusting parameters such as the deposition potential and electrolyte concentration, the properties of the heterostructure can be precisely tuned [42,43].

Economy is another distinctive feature of this method. Electrochemical deposition is more cost-effective than other methods, requiring more straightforward equipment and resources [44,45]. This makes it suitable for academic research and favorably positions it for industrial applications. The ability of this method to achieve consistent uniform deposition over large areas is invaluable, significantly when heterogeneity could compromise performance [46,47].

The thermal annealing method, particularly in forming multilayer heterostructures, has its clear advantages [48,49]. Annealing in a precursor-rich environment promotes the formation of well-defined phases, enhancing the crystallinity and phase purity of the heterostructures [50,51]. This, in turn, leads to improved optical, electrical, and mechanical properties, making the heterostructures more suitable for a range of applications [52,53]. Oxygen annealing, in turn, aids in the removal of unwanted impurities or defects that may have been introduced during the deposition process [54,55,56,57]. Correcting such flaws can significantly enhance the stability and longevity of the heterostructure’s performance [58,59]. Additionally, the process allows for precise tuning of the stoichiometry of oxide layers, allowing researchers to modulate and optimize the properties of the heterostructure according to specific application needs [60].

In the quest for effective and economically viable methods of depositing CdO on semiconductor surfaces, our proposed electrochemical deposition method with oxygen annealing stands out as a promising approach. Utilizing the advantages of both methods, we propose a pathway to obtaining high-quality CdO/CdS/ZnO heterostructures that are efficient, scalable, and accessible for broader applications in solar energy. When applied with electrochemical deposition, oxygen annealing can offer a comprehensive approach to forming oxide heterostructures, ensuring both deposition precision and enhancement of material properties.

## 2. Materials and Methods

### 2.1. Sample Preparation

Monocrystalline ZnO was chosen as the substrate for the growth of CdS films. Samples were cut into plates measuring 1 cm × 2 cm × 0.2 cm and polished on both sides. Simple chemical etching in an aqueous solution of HCl (5% by mass) was used for sample cleaning. Following this, the samples were rinsed in alcohol and vinegar. Immediately after rinsing, the samples were moved to the experiment to prevent oxidation of the prepared surface and dirt deposition.

### 2.2. Electrolyte Preparation

The electrolyte was prepared by dissolving cadmium sulfate (CdSO_4_, 0.05 M) and sodium sulfide (Na_2_S, 0.05 M) into deionized water. To achieve these concentrations specifically, 0.05 moles of CdSO_4_ and 0.05 moles of Na_2_S were dissolved in a total volume of 1 L of deionized water, using a magnetic stirrer for uniform mixing. This solution was prepared and maintained at a temperature of 85 °C for 1 h on a hot plate to ensure complete dissolution of the reagents and proper interaction between them. Subsequently, sodium perchlorate (NaClO_4_) was added to the solution to achieve a concentration of 0.5 M as a supporting electrolyte. For this, 0.5 moles of NaClO_4_ were added to the previously prepared 1-L solution, ensuring the final volume was adjusted accordingly. All reagents were purchased from the company Spezprompostach, Dnipro, Ukraine.

### 2.3. Devices

The primary method chosen to prepare CdO/CdS was electrochemical deposition followed by oxygen annealing. The electrochemical cell for electrochemical deposition had two electrodes—a working electrode where the deposition occurs and a counter electrode. The working electrode was the mono-ZnO plate, and the counter electrode was a platinum (Pt) plate. The cell also contained a silver/silver chloride reference electrode to control the working electrode’s potential.

Annealing was carried out in a JetFirst furnace (Jipelec, Montpellier, France), which was chosen because it provides uniform heating and precise temperature control.

### 2.4. Electrochemical Deposition

Electrochemical deposition occurred at a constant applied potential of 0.8 V for 1 h. The hypothesis was that such a regime would ensure the formation of a continuous film rather than crystallite grains, as usually occurs [61,62].

The mechanism of electrochemical deposition can be simplified as follows. Cd^2+^ ions in the electrolyte are reduced at the cathode, forming Cd, which then reacts with S^2−^ ions to form CdS. The electrochemical deposition of CdS on the ZnO surface involves the reduction of cadmium ions (from CdSO_4_) and sulfide ions (from Na_2_S) on the ZnO electrode surface, forming a CdS layer. The overall scheme of this process can be represented as follows.

In the electrolyte, salts dissociate into their respective ions upon dissolution in the solvent (typically water):

CdSO_4_ → Cd^2+^ + SO_4_^2−^ (1)

Na_2_S → 2Na^+^ + S^2−^ (2)

At the cathode (ZnO electrode), cadmium and sulfide ions are reduced and precipitate in the form of CdS:

Cd^2+^ + S^2−^ → CdS (3)

During the process, oxidation occurs at the anode to maintain charge neutrality.

Thus, the overall electrochemical reaction can be represented as follows:

Cd^2+^ + S^2−^ + 2H_2_O → CdS + O_2_ + 4H^+^ (4)

### 2.5. Post-Treatment of CdS Film

Given the task of growing a continuous, uniform film on the ZnO surface, it was necessary to consider that such films often crack, disintegrate, and exhibit significant stress due to the mismatch of crystal lattices between the substrate and the deposited film [63,64]. Also, poor adhesion and gradients in local concentrations are possible [65], leading to additional defects at the interface of the two compounds [66,67].

A potential solution to these issues is additional chemical or electrochemical etching. This approach can help address imperfections, surface irregularities, and stresses in the deposited film, potentially improving the film’s crystallinity and overall properties.

Therefore, electrochemical etching was performed in a 5% hydrochloric acid solution. A standard regime at a constant potential of 2 V was applied. The etching was conducted at room temperature in darkness for 10 min. To ensure the removal of reaction products from the sample surface, the electrolyte was stirred with a Teflon stirrer.

Afterward, the sample was removed from the solution, rinsed in deionized water, and proceeded to the annealing process.

### 2.6. Formation of CdO Layer on CdS/ZnO Surface

The thin CdS film was placed in a JetFirst furnace preheated to a constant temperature of 400 °C and annealed in an oxygen atmosphere for 1.5 h. The samples were allowed to cool down to room temperature inside the furnace after the annealing process. This facilitated a controlled cooling process to ensure the integrity of the CdO/CdS phases.

Annealing the cadmium sulfide (CdS) film in an oxygen atmosphere leads to the formation of cadmium oxide (CdO) as cadmium sulfide reacts with oxygen to form cadmium oxide and sulfur dioxide [68]:

2CdS + 3O_2_ → 2CdO + 2SO_2_↑ (5)

The high temperature facilitated the conversion of deposited precursors into well-crystallized CdO/CdS phases. The oxygen atmosphere played a crucial role in ensuring the formation of oxide phases free from unwanted impurities or defects.

**Figure 1:**
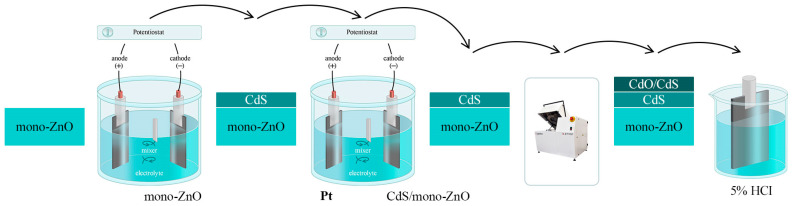
Scheme of formation of CdO/CdS/ZnO heterostructure.

Subsequently, the sample was removed from the furnace and chemically etched (without applying potential) in an HCl solution for 3 min to remove the top layer and unwanted reaction products from the sample surface. The overall scheme of forming the CdO/CdS/ZnO heterostructure is illustrated in Figure 1.

### 2.7. Characterization

Scanning electron microscopy (SEM) was utilized for morphological analysis, employing the SEO-SEM Inspect S50-B microscope (Sumy Plant of Electronic Microscopes, Sumy, Ukraine) at a voltage of 20 kV. The deposited layers were examined using energy-dispersive X-ray (EDX) spectroscopy for compositional analysis and material identification. Spectra acquisition modes included point analysis and surface mapping technology (20 kV). X-ray diffraction (XRD) was conducted for structural analysis using the Dron-3 M system (Sumy State University, Sumy, Ukraine) with unfiltered Cu Ka radiation in the angle range of 2θ from 10° to 80° with a step of 0.01°. Raman measurements were carried out at room temperature using the RENISHAW inVia Reflex system (Renishaw plc, Wotton-under-Edge, UK). Luminescence measurements were performed on a miniature DAC from easyLab, placed in a continuous-flow cryostat CF 200 Oxford Instruments with an ITC4 Oxford Instruments temperature controller (easyLab Technologies Ltd., now part of Oxford Instruments, Abingdon, UK). Luminescence was collected in a backscattering geometry using a Yobin Yvon-Spex Triax 320 monochromator equipped with a Spectrum One CCD camera (Horiba Jobin Yvon, Longjumeau, France). This experiment’s luminescence was excited using 405 nm radiation from a 100 mW diode laser or the 325 nm line from a 20 mW He–Cd laser.

## 3. Results and Discussion

### 3.1. SEM Analysis

**Figure 2:**
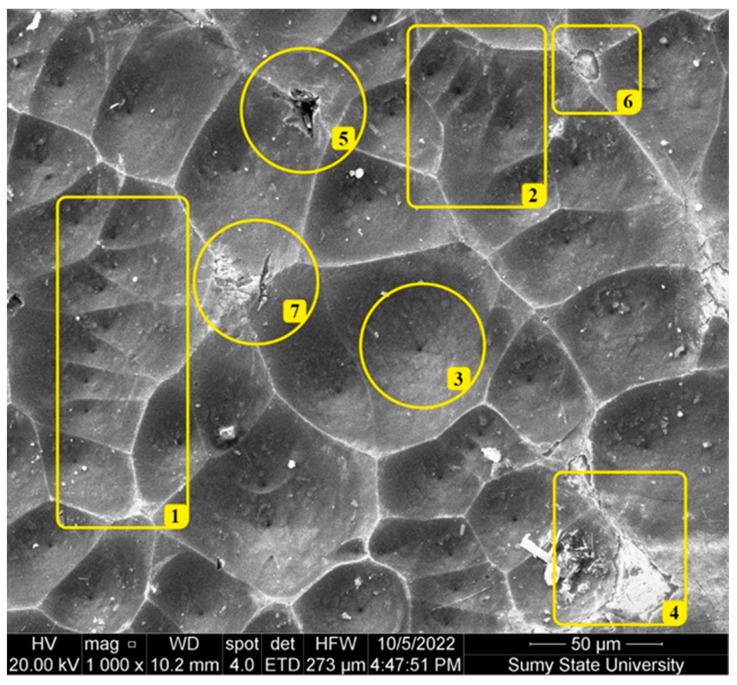
SEM images of the surface of the CdO/CdS/ZnO heterostructure.

Figure 2 presents the surface morphology of the synthesized CdO/CdS/ZnO heterostructure. The characteristic areas highlighted in yellow are interesting for understanding the surface relief. Overall, the analysis of the macro-morphology of the surface reveals the presence of shallow crater-like depressions separated by thin archipelagos. These craters vary in shape and size, typically forming three- or five-sided etching figures. The cross-sectional diameter of the craters ranges from 10 to 80 µm. For instance, in area 1, almost parallel triangular craters can be seen, each containing a pore in the center. Pores are also observed in larger craters, for example, in area 2 of Figure 2. Generally, the surface macro-morphology, formed by craters with a pore inside, resembles Voronoi diagrams. Area 3 in Figure 2 shows one of the largest craters captured in the microscope’s field of view. It can be observed that the pore in this crater has radial rays. The size of the pore is about 1 µm. The crater’s surface has a fluffy structure consisting of flakes and microparticles. The size of the microparticles varies in the range of 50–200 nm. The inter-crater walls have the appearance of archipelagos with sharp peaks, which in cross-section are approximately 50–100 nm wide and up to 100 µm in length. At places where these archipelagos intersect, etching of the peaks can be seen (areas 4, 5, 6, and 7 in Figure 2). Such behavior can be described by the mechanism of electrochemical breakthrough during the electrochemical processes of surface treatment of the crystal.

**Figure 3:**
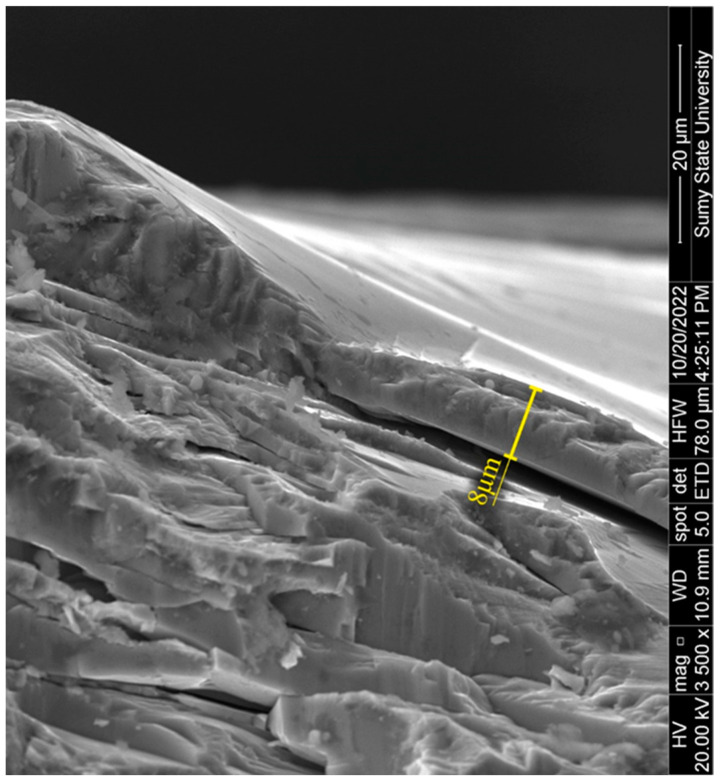
SEM images of the cross-section of the CdO/CdS/ZnO heterostructure.

Figure 3 presents the cross-section of the film. SEM analysis determined that the film thickness varies in the range from 7 to 15 µm, highlighting the unevenness of its formation. In the thinnest area, it loosely adheres to the substrate, indicating potential delamination of the film, which requires further investigation to determine its stability and potential impact on the device’s efficiency.

Overall, such a developed morphology indicates a complex film formation process associated with electrochemical deposition and oxygen annealing. Secondly, it could enhance solar cells’ efficiency as the structure provides a larger surface area for light absorption.

As revealed in our analysis, the distinctive surface macro-morphology characterized by crater-like depressions and thin archipelagos plays a crucial role in the photovoltaic performance of CdO/CdS/ZnO heterostructures. These features are not merely topographical; they contribute significantly to the material’s light absorption and scattering properties, which are essential for solar energy applications.

The presence of shallow crater-like depressions interspersed with archipelago-like structures increases the surface area available for light absorption. The varying shapes and sizes of these depressions, particularly the three- or five-sided etching figures, can enhance light-trapping capabilities by scattering incident light into deeper layers of the material, thereby increasing the likelihood of photon absorption. This phenomenon can lead to a more efficient generation of charge carriers, which is pivotal for the conversion of solar energy into electricity.

Furthermore, as observed in our SEM analysis, the unique topology characterized by craters with a central pore and radial rays introduces additional pathways for light to interact with the material. The pores and their associated radial rays may act as focal points for light concentration, enhancing local absorption and potentially increasing the photogenerated current density in these regions.

However, the uneven film thickness and potential for delamination observed in the thinnest areas of the film highlight a limitation of the current synthesis approach. These areas may act as sites of reduced mechanical stability, posing a risk to the long-term durability and efficiency of the solar cells. The observed delamination could create discontinuities in the conductive pathways, adversely affecting the overall charge collection efficiency of the device.

In response to these findings, further optimization of the electrochemical deposition and oxygen annealing processes is necessary to minimize film unevenness and prevent delamination. By refining these synthesis parameters, a more uniform film thickness may be achieved while preserving the advantageous surface features that contribute to enhanced light absorption.

### 3.2. EDX Analysis

**Figure 4:**
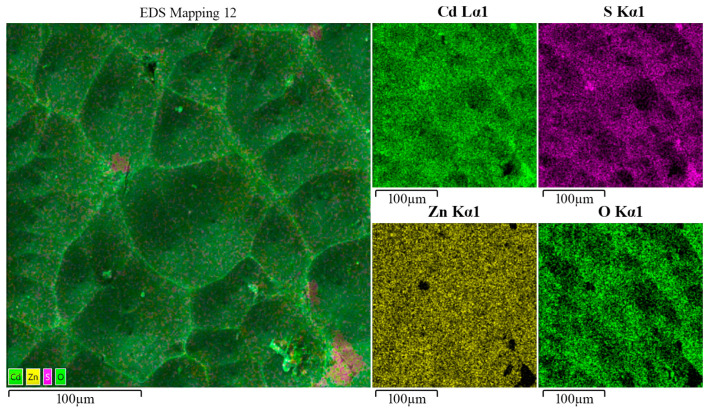
EDX analysis of the sample surface, conducted using mapping technology.

Energy-dispersive X-ray (EDX) analysis of the CdS films was conducted using a mapping technique. The EDX analysis was performed at an accelerating voltage of 20 kV to optimize the resolution and sensitivity for the elemental mapping of Cd, S, Zn, and O on the sample surface. This mapping allowed for a detailed study of the chemical composition of the film in different areas (Figure 4). Based on the data obtained, it is evident that the elements Cd and S are uniformly distributed across the surface.

Oxygen was present across the entire area under investigation, except at the bottom of the pores. This could indicate that the annealing process in an oxygen atmosphere effectively enhanced the films’ crystallinity. However, the absence of oxygen at the bottom of the pores may suggest the need for further optimization of this process to ensure a more uniform distribution of oxygen. A small amount of zinc was detected in the film, concentrated at the tops of the interpore walls. This could indicate zinc atom migration from the ZnO substrate to the CdS film during the annealing process or etching of the film at the peaks of the archipelago during the final stages of sample processing.

**Figure 5:**
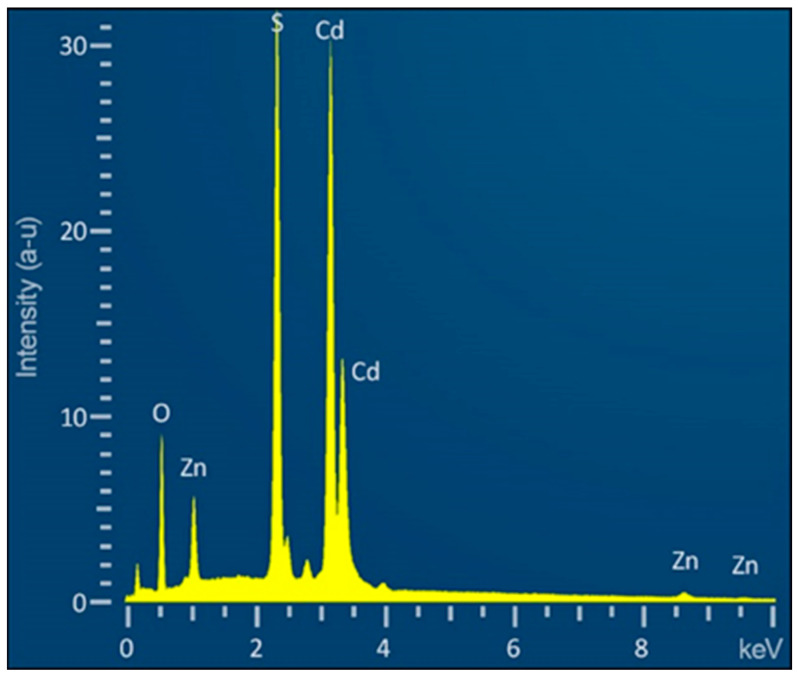
EDX analysis of the sample surface was conducted using mapping technology.

Figure 5 and Table 1 present the EDX analysis conducted at a specific point on the sample.

The surface predominantly comprises S atoms (40.69%) and Cd (37.39%). Zinc (Zn) constitutes a relatively minor 3.69%, suggesting a lesser concentration in the area under investigation, likely a residual component from the underlying ZnO plate.

The dominance of sulfur in the composition correlates with the significant presence of the CdS layer in the heterostructure. The high atomic percentage of Cd indicates its substantial role in forming the CdS and CdO layers. The almost uniform distribution of these elements suggests a uniform CdS film, crucial for stable optical and electronic properties across the entire surface.

The 18.23% oxygen content is significant, resulting from the formation of CdO during the annealing process in an oxygen atmosphere. However, the aforementioned absence of oxygen in the lower parts of the pores indicates that further optimization of the annealing process is required to achieve a more uniform oxygen distribution throughout the structure. Such uniformity is crucial for ensuring consistent material properties across the entire heterostructure.

### 3.3. XRD Analysis

**Figure 6:**
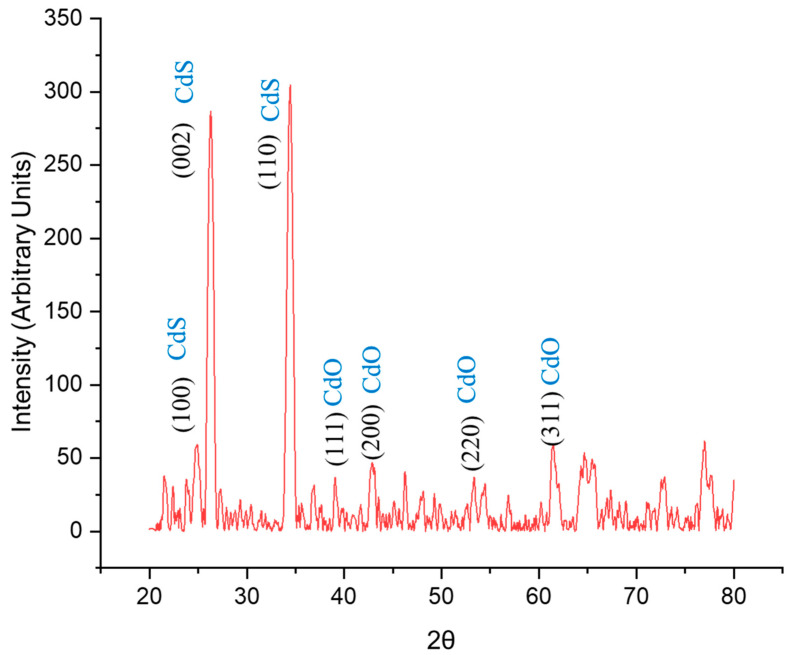
XRD spectrum of CdO/CdS/ZnO heterostructure.

In the X-ray diffraction (XRD) analysis of the CdO/CdS/ZnO heterostructure, observed peaks present a complete picture of the material’s crystalline structure, with several peaks associated with different compounds in the heterostructure (Figure 6). Notably, the peaks at 24.85° and 26.30° correspond well with the (100) and (002) planes of hexagonal CdS (JCPDS Code: 41-1049), respectively, indicating a significant presence of this phase with high crystallinity. The peak at 34.45°, slightly shifted from the typical position for the (110) plane of hexagonal CdS, suggests some lattice distortion, potentially due to interaction with other phases in the heterostructure.

Conversely, the peaks at 39.05°, 42.95°, 54.50°, 63.1°, and 65.7° indicate the presence of cubic CdO (JCPDS: 05-0640). However, these peaks show deviations from the standard positions expected for cubic CdO, suggesting that CdO in the heterostructure is likely in a polycrystalline phase with a high degree of amorphization. This conclusion is drawn from these peaks’ broad and slightly shifted nature, characteristic of materials with a high degree of structural disorder. Such amorphization in CdO could arise from deposition conditions, interaction with CdS and ZnO layers, or intrinsic defects within the CdO structure.

While less defined, the peak at 77.0° could be associated with higher-order reflections from CdS or CdO, further complicating the structural interpretation. The absence of distinct ZnO peaks in the XRD spectrum suggests that the overlying CdO and CdS layers effectively mask the ZnO substrate. This observation aligns with the surface morphology and composition data obtained from scanning electron microscopy (SEM) and energy-dispersive X-ray spectroscopy (EDX), indicating complete coverage of the ZnO substrate by CdO/CdS layers.

Also, the spectrum reveals weak peaks that are not precisely identified with CdO or CdS, which could indicate the presence of impurities or additional crystalline phases. Considering the synthesis method and the materials used, these additional peaks could be attributed to an intermediate phase formed between CdO and CdS or due to interaction between the ZnO substrate and the deposited layers. Phases such as zinc cadmium sulfide (Zn_x_Cd_1−x_S) or zinc cadmium oxide (Zn_x_Cd_1−x_O) might form during the deposition or annealing process, especially under conditions that facilitate mutual diffusion of ions between layers. Due to their unique lattice parameters, these phases could exhibit diffraction peaks at angles slightly different from the pure phases of CdO, CdS, and ZnO.

In summary, the XRD spectrum of the CdO/CdS/ZnO heterostructure reveals a complex interplay of crystalline and amorphous phases. The dominance of well-crystallized hexagonal CdS is evident. At the same time, the CdO phase exhibits signs of polycrystallinity and amorphization, likely due to the deposition process and its interaction with other layers in the heterostructure. The observed peak shifts and broadening in the X-ray diffraction pattern are characteristic of polycrystalline films where structural features like grain boundaries, defects, and lattice mismatches play a significant role [69,70]. These structural properties are especially prevalent in films synthesized under conditions that favor interphase diffusion and interaction, such as electrochemical deposition followed by annealing [71,72]. In conclusion, the XRD analysis of the CdO/CdS/ZnO heterostructure underscores the formation of a composite material with integrated cubic CdO and hexagonal CdS phases, demonstrating lattice deformation and structural complexity.

### 3.4. Raman Analysis

**Figure 7:**
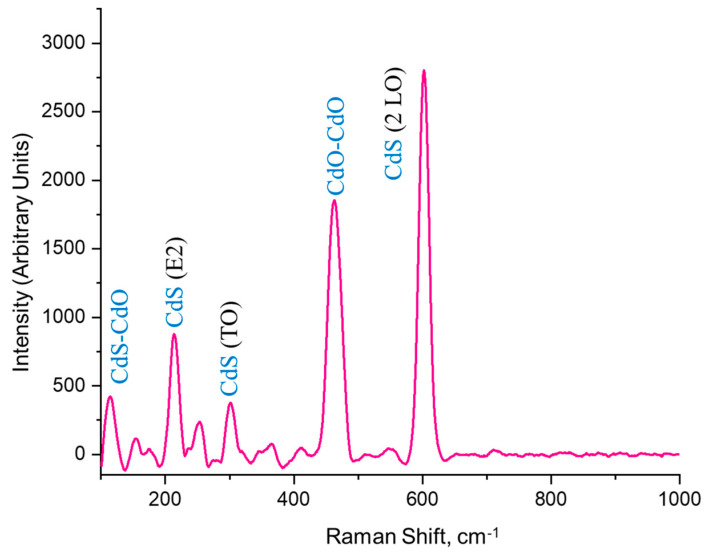
Raman spectrum of the CdO/CdS/ZnO heterostructure.

Raman measurements were conducted at room temperature using the RENISHAW inVia Reflex Raman microscope, equipped with a 514 nm argon-ion laser as the excitation light source. The Raman scattering spectrum of the CdO/CdS/ZnO heterostructure presents a complex interaction of vibrational modes, attributable to the various contributions of CdO and CdS, with the ZnO substrate likely not contributing significantly due to its overshadowing by the CdO/CdS layers (Figure 7).

The most intense peak observed at 601 cm^−1^ in the Raman scattering spectrum is characteristic of the longitudinal optical (LO) phonon modes of CdS in its hexagonal wurtzite structure. This peak typically manifests in this frequency range due to the vigorous Raman activity of the LO phonon in CdS [73]. The prominence of this peak indicates a significant contribution of the CdS layer to the heterostructure, possibly reflecting a high degree of crystallinity and well-defined phonon modes in the CdS layer.

Following this, the second most intense peak at 464 cm^−1^ can be attributed to the vibrational modes of CdO [74]. In the cubic rock salt structure of CdO, the active Raman scattering modes are less pronounced than in CdS, but they typically appear in this spectrum area [75]. This peak may arise from optical phonons associated with lattice vibrations of CdO, reflecting the presence of the crystalline component of CdO in the heterostructure.

The third intense peak at 214 cm^−1^ indicates the high-phonon mode E2 of hexagonal CdS. This mode is associated with sulfur atom vibrations in the lattice and is indicative of the hexagonal crystalline structure of CdS [76]. Its presence further confirms the crystalline nature of CdS in the heterostructure and indicates a well-ordered lattice.

Lower intensity peaks at 115 cm^−1^ and 303 cm^−1^ also provide valuable information. The peak at 115 cm^−1^ could be associated with second-order combination scattering or multiphonon processes in CdO–CdS. Such low-frequency modes often indicate lattice defects in the crystalline structure. Meanwhile, the peak at 303 cm^−1^ could correspond to the TO phonon mode of CdS or a second-order phonon process in CdO. The lower intensity of this peak compared to the LO mode is typical due to the generally weaker Raman activity of TO modes in these materials [77].

Since ZnO is a substrate that overlaps features with CdO and CdS in the Raman scattering spectrum, its specific contributions are likely masked. ZnO typically exhibits a solid E2 mode around 438 cm^−1^ and a weaker low E2 mode. Still, these are likely overshadowed by the more dominant characteristics of CdO and CdS in the heterostructure.

### 3.5. PL Analysis

Luminescence measurements were performed using a miniature DAC from easyLab, housed in an Oxford Instruments CF 200 continuous-flow cryostat with an ITC4 Oxford Instruments temperature controller, enabling precise control over the measurement environment. The temperature during the photoluminescence (PL) measurements was maintained at a specific value within 300 K. Luminescence was excited using either 405 nm radiation from a 100 mW diode laser or the 325 nm line from a 20 mW He–Cd laser and collected in a backscattering geometry using a Yobin Yvon-Spex Triax 320 monochromator equipped with a Spectrum One CCD camera.

**Figure 8:**
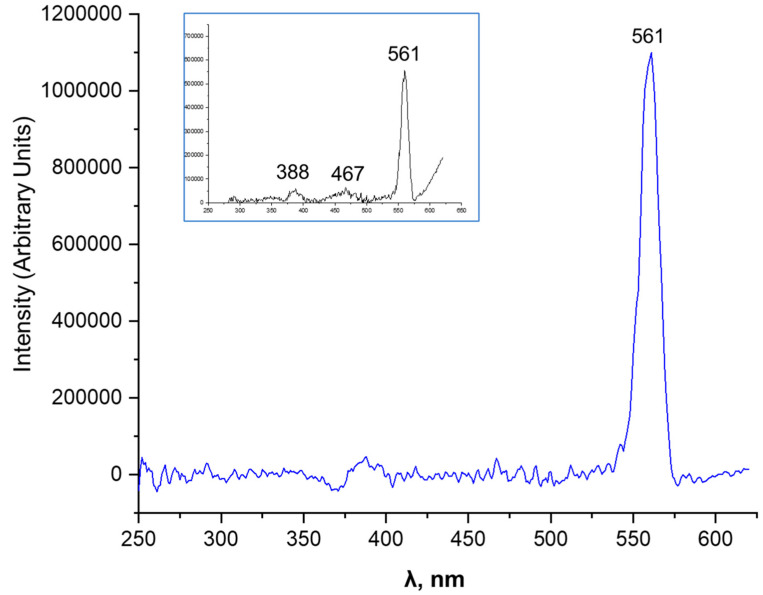
PL spectrum of the CdO/CdS/ZnO heterostructure and excitation spectrum (inset).

The photoluminescence (PL) spectrum of the CdO/CdS/ZnO heterostructure exhibits a prominently expressed peak at 561 nm, falling into the green–yellow region of the visible spectrum, accompanied by a notably narrow full width at half maximum (FWHM) of 13.52449 nm (Figure 8). This narrow FWHM indicates a uniform distribution of luminescent centers, implying high crystalline quality in the heterostructure.

The dominant contribution to this emission peak is attributed to the CdS component, particularly the hexagonal CdS phase, known for its strong luminescence due to band-edge transitions in this wavelength range [78]. However, the observed peak wavelength is slightly red-shifted compared to the typical band-edge emission of bulk cadmium, which typically lies between 475 and 520 nm [79]. This redshift can be explained by several interrelated factors inherent to the unique composition and structure of the heterostructure.

First, quantum confinement effects in nanostructured materials such as our CdO/CdS/ZnO heterostructure can shift the emission peak. When the size of CdS crystallites approaches the scale of the Bohr radius of the exciton, this quantum confinement can significantly change the electronic and optical properties, manifesting as shifts in the emission spectrum. In addition, the lattice strain in the heterostructure arising from the mismatch between the cubic CdO, hexagonal CdS, and ZnO components contributes to this shift. Deformation-induced band structure modifications of CdS affect its optical properties, leading to the observed redshift in the PL spectrum. In addition, defect states in CdS, such as vacancies or interstitials, can introduce localized states within the band gap, leading to emission at longer wavelengths. These defect-related emissions are crucial for determining the overall optical behavior of semiconductor heterostructures.

Heterojunction effects at the interface between CdO, CdS, and ZnO also play a significant role in the optical properties of the heterostructure. The interaction between different materials at these junctions can introduce new energy levels or change existing ones, affecting the shift of the PL peak.

In addition to the PL spectrum, the excitation spectrum measured with an excitation monochromator set at 450 nm shows a peak at 561 nm, which corresponds to the emission peak. This correspondence indicates that the observed photoluminescence is mainly due to the CdS phase in the heterostructure. The shift of the emission peak from typical values for bulk CdS, combined with the homogeneity of the size distribution of the emitting centers, as implied by the narrow FWHM, is a crucial feature for optoelectronic applications. The corresponding peak positions in the photoluminescence and photoluminescence excitation (PLE) spectra confirm the relevance of this wavelength in the optical properties of the heterostructure, highlighting its potential utility in devices such as LEDs or photodetectors.

Overall, the interplay of quantum confinement, lattice strain, defect states, and heterojunction effects shape the observed optical behavior of the CdO/CdS/ZnO heterostructure. Additionally, two extended low-intensity peaks at 388 nm and 467 nm are observed in the excitation spectrum. The appearance of these peaks can be rationalized by considering several factors intrinsic to the material composition and its interfacial properties.

The peak at 388 nm, falling in the ultraviolet region of the spectrum, is likely attributed to the ZnO component of the heterostructure. ZnO is known for its ultraviolet solid emission [80], typically centered around this wavelength due to band edge transitions. The presence of this peak in the excitation spectrum indicates that ZnO plays a role in absorption and subsequent emission processes in the heterostructure. The relatively low intensity of this peak suggests that the CdO/CdS film densely covers the substrate surface yet is sufficiently transparent to allow the ZnO peak to emerge.

The peak at 467 nm, located in the blue region of the spectrum, could be associated with emissions related to defects in the heterostructure. In semiconductor materials like CdS and ZnO, defects such as vacancies or impurities can create localized energy states within the band gap. These states can facilitate transitions emitting light at wavelengths different from band-edge emissions. The blue emission at 467 nm may indicate such defect-related transitions in the CdS or ZnO components or at the interfaces between different layers in the heterostructure.

These additional peaks also underscore the complex interaction of different materials in the heterostructure. Interfaces between CdO, CdS, and ZnO can introduce additional energy states or alter existing ones, leading to emissions at various wavelengths [81,82]. The peak at 467 nm, in particular, could be influenced by heterojunction effects, where the interaction between different semiconductor materials may lead to new optical transitions.

## 4. Discussion

The synthesis and characterization of CdO/CdS/ZnO heterostructures as detailed in our study highlight significant avenues for enhancing solar cell performance. These heterostructures, through their unique structural and optical properties, offer multiple pathways for improving the efficiency of solar energy conversion systems. Firstly, the heterostructure design capitalizes on the synergistic properties of CdO, CdS, and ZnO. It combines their strengths to address common limitations in solar cells, such as inefficient light absorption and charge carrier recombination.

A CdS layer is particularly beneficial for solar cells due to its favorable bandgap (~2.4 eV), which is well-suited for absorbing visible light [83,84]. When coupled with CdO, which has a smaller bandgap, a broader spectrum of solar radiation can be harnessed, from UV to visible light, thereby improving the overall light absorption efficiency of the heterostructure. Furthermore, the interface between the CdS and ZnO layers can form a type II heterojunction, facilitating efficient charge separation and reducing recombination losses. This heterojunction ensures that photogenerated electrons and holes are effectively separated and directed toward their respective electrodes, thereby increasing the photocurrent and, consequently, the solar cell’s efficiency.

Additionally, the surface morphology of the synthesized heterostructures, characterized by crater-like formations and varying sizes, enhances light trapping. This morphology increases the effective surface area for light absorption and induces scattering within the solar cell, further improving light-harvesting capabilities. The porous nature of these structures also favors the diffusion of electrolytes in photoelectrochemical cells, enhancing the interface reactions crucial for solar energy conversion.

Moreover, the high crystalline quality of the CdS phase, as evidenced by the PL analysis, indicates fewer defects that would otherwise act as recombination centers for charge carriers. This high crystalline quality is essential for maintaining high charge carrier mobility and minimizing energy losses, contributing to higher conversion efficiencies.

In light of these findings, future research should aim to further refine the synthesis techniques to enhance the uniformity and structural stability of these heterostructures. Optimizing the thickness and composition of each layer to tailor the absorption properties and charge carrier dynamics could lead to solar cells with significantly improved performance. Moreover, exploring the integration of these heterostructures into existing solar cell architectures could offer practical pathways for their deployment in next-generation solar technologies.

Incorporating CdO/CdS/ZnO heterostructures into solar cells could revolutionize solar energy technology by providing more efficient, cost-effective solutions. By harnessing a broader spectrum of solar radiation and improving charge separation and transport, these heterostructures can significantly increase solar cells’ efficiency, moving us closer to achieving sustainable, renewable energy sources for future generations.

As a logical continuation of this work, we have planned experiments related to the radiation modification of the heterostructures considered in this work. Fast, heavy, and light ions are used as irradiation sources. We have already successfully demonstrated their usefulness earlier [85,86,87]. After appropriate data analysis, the results will be presented in a following article.

## 5. Conclusions

In this study, we have proposed an effective method for synthesizing CdO/CdS/ZnO heterostructures, leveraging the combined electrochemical deposition and oxygen annealing techniques. The comprehensive analyses conducted using various techniques, including scanning electron microscopy (SEM), energy-dispersive X-ray (EDX) spectroscopy, X-ray diffraction (XRD), and photoluminescence (PL) spectroscopy, have yielded insightful results regarding the structural, compositional, and optical properties of the synthesized heterostructures.

The SEM analysis reveals a complex surface macro-morphology characterized by crater-like formations with varying sizes, indicating a detailed interplay in the heterostructure formation process. The uniform distribution of Cd and S elements across the surface, as evidenced by EDX mapping and the absence of significant contributions from the ZnO substrate, reflects the effective masking by the overlying CdO and CdS layers. This observation is further corroborated by the XRD analysis, which shows peaks indicative of both hexagonal CdS and cubic CdO phases, suggesting a polycrystalline structure with high degrees of crystallinity and amorphization. Additionally, Raman analysis provided insights into vibrational modes and structural interactions within the heterostructure, further elucidating the complex interplay of its crystalline and amorphous phases, which is essential for optimizing its optoelectronic properties.

The PL analysis provided valuable insights into the heterostructure’s optical properties. The emission peak at 561 nm and its corresponding narrow FWHM emphasize the high crystalline quality and uniformity of luminescent centers, primarily attributed to the hexagonal CdS phase. The observed redshift in the emission peak wavelength and additional low-intensity peaks in the excitation spectrum reflect the complex structural and optical interplay within the heterostructure, possibly influenced by quantum confinement, lattice strain, defect states, and heterojunction effects.

The synthesis method and the properties of the resulting heterostructure point towards promising future research prospects and potential applications, particularly in solar energy. The distinct structural features and the high crystalline quality of the heterostructure suggest its suitability for enhancing the efficiency of solar cells. The varied morphology, combined with the adequate absorption and emission properties as evidenced in the PL analysis, indicates potential for application in optoelectronic devices like LEDs and photodetectors. The presence of both CdS and CdO phases in the heterostructure could be harnessed to optimize light absorption and conversion efficiencies in solar cells.

Further research could focus on optimizing the synthesis process to achieve even more excellent uniformity in the distribution of components and enhance the structural stability of the heterostructure. Understanding and controlling the interface interactions between CdO, CdS, and ZnO could further improve the optical and electronic properties, which are crucial for developing high-efficiency solar energy devices.

In conclusion, the synthesized CdO/CdS/ZnO heterostructures, with their intricate structural and optical characteristics, present a significant potential for advancing solar energy technologies. The comprehensive characterization of these heterostructures lays the groundwork for future explorations and optimizations, paving the way for their application in efficient, sustainable solar energy solutions.

## Figures and Tables

**Table 1:** Compositional analysis of the CdO/CdS/ZnO heterostructure surface by EDX.

Element	Compound, At. %
S	40.69
Cd	37.39
O	18.23
Zn	3.69

## Data Availability

Data are contained within the article.
